# Valorization of *Adhatoda vasica* leaves: Extraction, *in vitro* analyses and *in silico* approaches

**DOI:** 10.3389/fnut.2023.1161471

**Published:** 2023-03-17

**Authors:** Mithun Rudrapal, Sugumari Vallinayagam, Sahar Aldosari, Johra Khan, Hind Albadrani, Alaa Al-Shareeda, Mehnaz Kamal

**Affiliations:** ^1^Department of Pharmaceutical Sciences, School of Biotechnology and Pharmaceutical Sciences, Vignan's Foundation for Science, Technology and Research (Deemed to be University), Guntur, India; ^2^Department of Biotechnology, Vel Tech Rangarajan Dr. Sagunthala R and D Institute of Science and Technology, Chennai, Tamil Nadu, India; ^3^Department of Medical Laboratory Sciences, College of Applied Medical Sciences, Majmaah University, Al Majma'ah, Saudi Arabia; ^4^Health and Basic Sciences Research Center, Majmaah University, Al Majma'ah, Saudi Arabia; ^5^Department of Cellular Therapy and Cancer Research, King Abdullah International Medical Research Center, Riyadh, Saudi Arabia; ^6^Department of the Saudi Biobank, King Abdullah International Medical Research Center, Riyadh, Saudi Arabia; ^7^Department of Pharmaceutical Chemistry, College of Pharmacy, Prince Sattam Bin Abdulaziz University, Al-Kharj, Saudi Arabia

**Keywords:** *Adhatoda vasica*, *in silico*, *in vitro*, pharmacological activities, vasicine

## Abstract

*Adhatoda vasica* (also called Vasaka) is a traditional medicinal herb used traditionally for the relief of cough, asthma, nasal congestion, bronchial inflammation, upper respiratory infections, bleeding disorders, skin diseases, leprosy, tuberculosis, diabetes, allergic conditions, rheumatism, tumor, and many more diseases. The present study aims to investigate the biological activities of vasicine, a potent alkaloid from A. vasica with different biological/ pharmacological assays and *in silico* techniques. Vasicine showed antimicrobial activity as evidenced fromthe colony-forming unit assay. It showed antioxidant activity in ABTS scavenging assay (IC_50_ = 11.5 μg/ml), ferric reducing power assay (IC_50_ = 15 μg/ml), DPPH radical scavenging assay (IC_50_ = 18.2 μg/ml), hydroxyl radical scavenging assay (IC_50_ = 22 μg/ml), and hydrogen peroxide assay (IC_50_ = 27.8 μg/ml). It also showed anti-inflammatory activity in proteinase inhibitory assay (IC_50_ = 76 μg/ml), BSA method (IC_50_ = 51.7 μg/ml), egg albumin method (IC_50_ = 53.2 μg/ml), and lipooxygenase inhibition assay (IC_50_ = 76 μg/ml). Vasicine showed antidiabetic activity in α-amylase inhibition assay (IC_50_ = 47.6 μg/ml), α-glucosidase inhibition assay (IC_50_ = 49.68 μg/ml), and non-enzymatic glycosylation of hemoglobin assay. It showed antiviral activity against HIV-protease (IC_50_ = 38.5 μg/ml). Vasicine also showed anticancer activity against lung cancer cells (IC_50_ = 46.5 μg/ml) and human fibroblast cells (IC_50_ = 82.5 μg/ml). *In silico* studies revealed that similar to the native ligands, vasicine also showed a low binding energy, i.e., good binding affinity for the active binding sites and interacted with α-amylase (-6.7 kcal/mol), α-glucosidase (-7.6 kcal/mol), cyclooxygenase (-7.4 kcal/mol), epidermal growth factor receptor (-6.4 kcal/mol), lipooxygenase (-6.9 kcal/mol), and HIV-protease (-6.4 kcal/mol). The present study ascertains the potential of vasicine as a bioactive compound isolated from A. vasica having therapeutic usefulness in many human diseases.

## 1. Introduction

*Adhatoda vasica* belongs to Acanthaceae family and known with many common names like as Vasaka, Baker or Malabar Nut (1). An evergreen plant with an average height of 1.0 to 2.5 m with a bitter taste and unpleasant smell (2). Many studies reported the use of leaves and flowers for curingasthma, cough, cold, expectorant, and antispasmodic. *In vivo* study on rats showed to prevent oxidative damage due to carbon tetrachloride (3). The phenolic compounds found in A.Vasica reported to scavenges the free radicals and displays highest antioxidant activity (4). These medical properties make *Adhatoda vasica* of immense interest to study its phytochemicals and active compounds for drug discovery.

**Figure 1:**
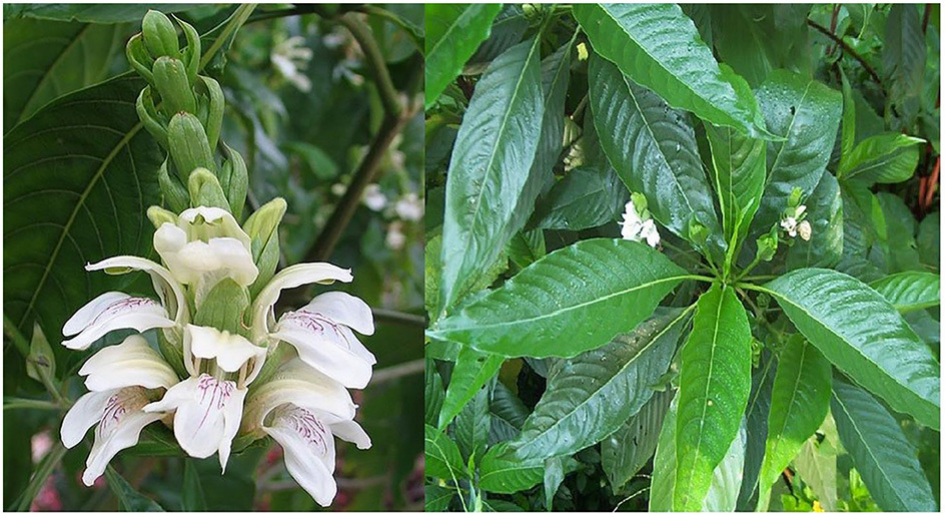
Flower and leaves of *Adhatodavasica*.

In the present day and age, herbal medicines have become more popular in the treatment of many diseases due to a popular notion that herbal medicines are safe with zero to very few side (adverse) effects (5, 6). *A. vasica* (Figure 1) is also called *Adhatodai* or *Vasaka* or *Arush*a (7). It is a recognized herbal remedy in Ayurvedic and Unani systems of medicine (6). It has been used in many traditional remedies for the management of various human diseases (6). The major chemical compounds of *A. vasica*belong to the quinozolinealkaloidal group. These quinozoline alkaloids are vasicine, and a bronchodilator alkaloid, vasicinone (7).

**Figure 2:**
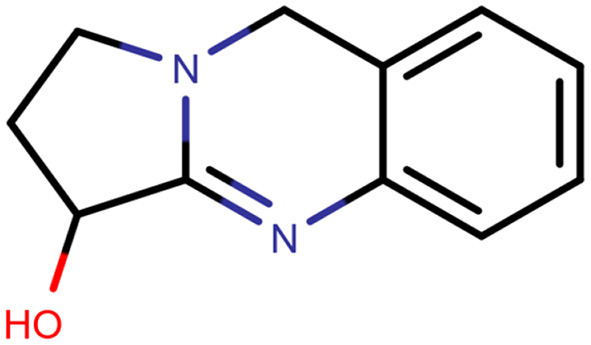
Chemical structure of vasicine.

Vasicine (Figure 2) is also called peganine (8). The bronchodilatory activity of vasicine is well reported. Bromhexine and ambroxol are the derivatives of vasicine, which are used as expectorants and mucolytics (9). Vasicine has been characterized by infrared spectroscopy, mass spectroscopy, nuclear magnetic resonance, and melting point. Identification of vasicine was donethroughspectral data comparison with those data that had been reported (10, 11).

People in ancient times used plant extracts as such as traditional herbal remedies but they were unable to find the individual compounds that are responsible for the biological effects (12). In this research, a double extraction system was used for the extraction and purification of vasicine. The primary extraction was carried out with a highly polar solvent (Soxhlet extraction) followed by a secondary extraction (column chromatography), which increases the specificity of the extraction process. Although column chromatography is generally used for purification, we have exploited its principle to optimize the solvent system. After extraction, we analyze the *in vitro* antibacterial activity, anti-inflammatory activity, anti-diabetic activity, and anti-oxidant activities. *In silico* techniques were used to study the interaction of vasicine with different target proteins used in the bioassay model.

## 2. Materials and methods

### 2.1. Collection of plant materials and processing

The fresh leaves of *A. vasica* were collected from the areas of Nagalapuram, Thoothukudi district, Tamil Nadu. Collected leaves were specifically separate out from other plant parts. Collected sample were washed with water to remove unwanted particles and dust. Leaves collected form *A. vasica* plants were authenticated by Dr. Srinivasan, Siddha Doctor, Government hospital, Nagalapuram, Thoothukudi, Tamil Nadu. Leaves of *A. vasica* were dehydrated and the size of the plant materials was reduced to moderate coarse powder. The dried plant materials were subjected to loss on drying test. The initial weight of leaves material was recorded. After drying, the leaves were weighed. This procedure was repeated until a constant weight was obtained. The powdered samples were sieved using sieving machine with mesh size 75 μ to achieve sample powder in even size. Small sized particle can release more extract so the 75 μ mesh sieved powder was preferred for the extraction process.

### 2.2. Extraction

The uniformly powdered samples were subjected to double extraction using Soxhlet extraction and column chromatographic extraction process to increase the quantity as well as to attain purity of extracted components from the plant materials of *A. vasica*. For the extraction process, solvents were selected based on their polarity (hexane, toluene, ethyl acetate, acetone, and methanol). In this method, 4 g of powdered substance was taken and 600 ml of solvent was used. The extraction process was carried out at the boiling point the solvent used for about 6–8 h and 6 cycles as preliminary extraction. All the chemical and solvents used were of analytical grade and were used as received without any further purification and were obtained from Sigma-Aldrich.

### 2.3. Qualitative confirmation

The qualitative analysis of the samples was done to verify the presence of alkaloids in the extract. Alkaloid tests, wavelength scan analysis and TLC analysis were performed for all eluted samples. Fourier-transform infrared spectroscopy (FT-TR) analysis was used to identify the type of functional groups present in vasicine for different fractions of the extract. Mayer's test and Wagner's test were carried out to qualitatively assess the presence of alkaloids in the extract (vasicine) (13–15).

### 2.4. UV spectroscopic analysis

The different fractions were collected from column chromatography (13) with different solvents and were analyzed with Hitachi, Spectrophotometer U-2800 (United Kingdom). The wavelength scan was carried out between 200 and 500 nm. The peaks obtained were compared with reported reference values to verify the presence of vasicine (281 nm) in the extract.

### 2.5. Thin layer chromatography

Approximately 10 μl of the sample was spotted on the completely dried TLC plate (7.5 × 2.5 cm, 0.5 mm thickness, silica gel G as stationary phase) and was placed in a beaker (developing chamber) previously saturated with the mobile phase (chloroform: methanol; 9:1 ratio). After the solvent raised to 3/4^th^ of the plate, the plates were taken out from the developing chamber and was visualizedin a UV chamber at 254 nm. On spraying the plate with Dragendroff's reagent, a prominent orange spot of vasicine was observed. The retention factor (R_f_) was also measured (16).

### 2.6. Fourier-transform infrared spectroscopic (FT-IR) analysis

Solid sample was preferred for the FT-IR analysis. The fractions obtained from column chromatography were analyzed using FT-IR (400 MHz Burker Advance spectrometer) to confirm the presence of the vasicine based on the functional groups present in it. The functional groups present in vasicine are O-H, C-H, C=N, C-N, C=C, and C-O groups. 10 μl/ 10 mg of sample was analyzed with the FT-IR instrument. The percentage transmittance (60–100%) vs. wave number (400–4,000 cm^−1^) was plotted and the peaks were viewed with software OPUS operator.

### 2.7. *In vitro* studies

#### 2.7.1. Antibacterial activity

Antibacterial assay was carried outby colony forming units (CFU) assay using anaerobic and facultative oral bacteria (17). Two different bacterial strains (*Escherichia coli* and *Bacillus badius)* were collected from NCCS, Pune. Pure cultures were sub cultured in Mueller-Hinton (MH) broth suggested by Bauer (18). The broth were kept in incubator at 37°C for 24 h. Two strains of Bacteria were grown in MH broth to an OD_600nm_ of 0.5. 2 μLaliquot of the bacteria and 5 ml of fresh MH broth (contains various concentration of vasicine) was added (19). The log_10_ reduction in CFU/ml was determined.

#### 2.7.2. Anti-oxidant activity

The anti-oxidant activity of vasicine was evaluated with ABTS [2,2′-azino-bis (3-ethylbenzothiazoline-6-sulfonic acid)] activity (20), ferric reducing power (FRAP) assay, DPPH radical scavenging activity, hydroxyl radical scavenging activity (21–24), and hydrogen peroxide assay (25).

#### 2.7.3. Anti-inflammatory activity

The anti-inflammatory activity of vasicine was investigated with lipooxygenase (LOX) inhibition assay (26), bovine serum albumin (BSA) method (27), egg albumin method (28, 29), and protein inhibitory action (30).

#### 2.7.4. Antidiabetic activity

The antidiabetic activity of vasicine was evaluated with *in vitro* enzymatic assays using α-amylase and α-glucosidase (31–36). The non-enzymatic glycosylation of hemoglobin assay was also carried out (37).

#### 2.7.5. HIV protease inhibition activity

The antiviral activity of vasicine against HIV protease was investigated using the HIV protease inhibition assay (38).

#### 2.7.6. Anticancer activity

The anticancer activity of vasicine for the potential treatment of lung carcinoma (A545) was evaluated in a multi-step process. Cytotoxicity of vasicine was investigated by cytotoxicity evaluation (human fibroblast cell line, C0135C) (39, 40), direct microscopic observation (41), and MTT assay (42).

### 2.8. *In silico* studies

MarvinSketch software was used to obtain the chemical structure of vasicine in “SDF” file format. Energy minimization of vasicine and conversion into “pdbqt” file format was carried out (43–45). The structure of α-amylase (PDB ID:4W93), α-glycosidase (PDB ID:3A4A), cyclooxygenase (PDB ID:5F1A), lipoxygenase (PDB ID:6N2W), HIV protease (PDB ID: 5KR0), and epidermal growth factor receptor (PDB ID:1IVO) were retrieved from the database (https://www.rcsb.org/) (46). Pre-processing of proteins for removal of side chains, identification of the active site, removal of heteroatoms, removal of water and addition of hydrogen atoms was carried out (43, 47–49). The coordinates of the active binding sites are as follows: α-amylase (x = −12.30, y = 4.25, z = −22.43), α-glycosidase (x = 21.31, y = −7.82, z = 23.30), cyclooxygenase (x = 41.74, y = 24.19, z = 239.73), epidermal growth factor receptor (x = 108.02, y = 66.26, z = 45.17), HIV protease (x = −16.70, y = 12.41, z = −20.16), and lipoxygenase (x = 42.34, y = 20.37, z = 36.35). Molecular docking was performed with the AutoDockVina to investigate the binding affinity of vasicine toward each target proteins. Discovery Studio Visualizer 2020 was used for the visualization of protein-ligand interactions (43, 50–53). The drug-likeness of vasicine was studied using Swiss ADME (54–57).

## 3. Results and discussion

### 3.1. Extraction and qualitative analysis

Five different solvent systems were used in the secondary extraction and different fractions were obtained from the column extraction process. Qualitative analyses such as wavelength analysis, TLC analysis, Mayer's test (58), and Wagner's test (59) were included to find the best solvent system for the extraction of vasicine. The phytochemical analysis results of vasicine are shown in Table 1.

**Table 1:** Qualitative alkaloid tests by Mayer's and Wagner's reagents for vasicine.

**Sl. No**.	**Solvent**	**Fractions**	**Mayer's test**	**Wagner's test**
1	Hexane	1	-	-
2	Toluene	1	-	-
		2	-	-
		3	-	-
		4	-	+
		5	-	+
3	Ethyl acetate	1	+	++
		2	++	++
		3	++	++
4	Acetone	1	++	+
		2	+	+
		3	+	+
5	Methanol	1	++	++
		2	+	+
		3	+	+

+, Present; ++, distinctively present; -, absent. Ethyl acetate fraction, acetone fraction, and methanol fraction showed positive results for alkaloid tests. However, the ethyl acetate fraction showed a more distinctive positive result. This was indicative that the alkaloid content in the ethyl acetate fraction was higher than the other fractions.

### 3.2. *In vitro* pharmacological evaluation

#### 3.2.1. Antimicrobial activity

**Figure 3:**
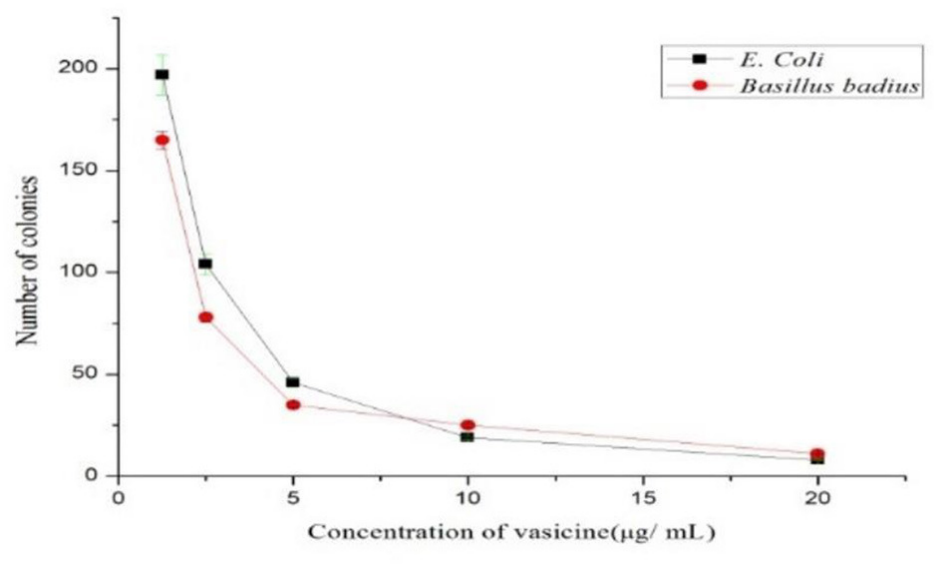
Anti-microbial Activity of vasicine for *E. coli* and *Bacillus badius*.

Based on the colony-forming unit assay technique, the antibacterial activity of vasicine was determined. The number of colonies reduced as the concentration of purified vasicine increased (Figure 3).

#### 3.2.2. Antioxidant activity

##### 3.2.2.1. ABTS scavenging activity

**Figure 4:**
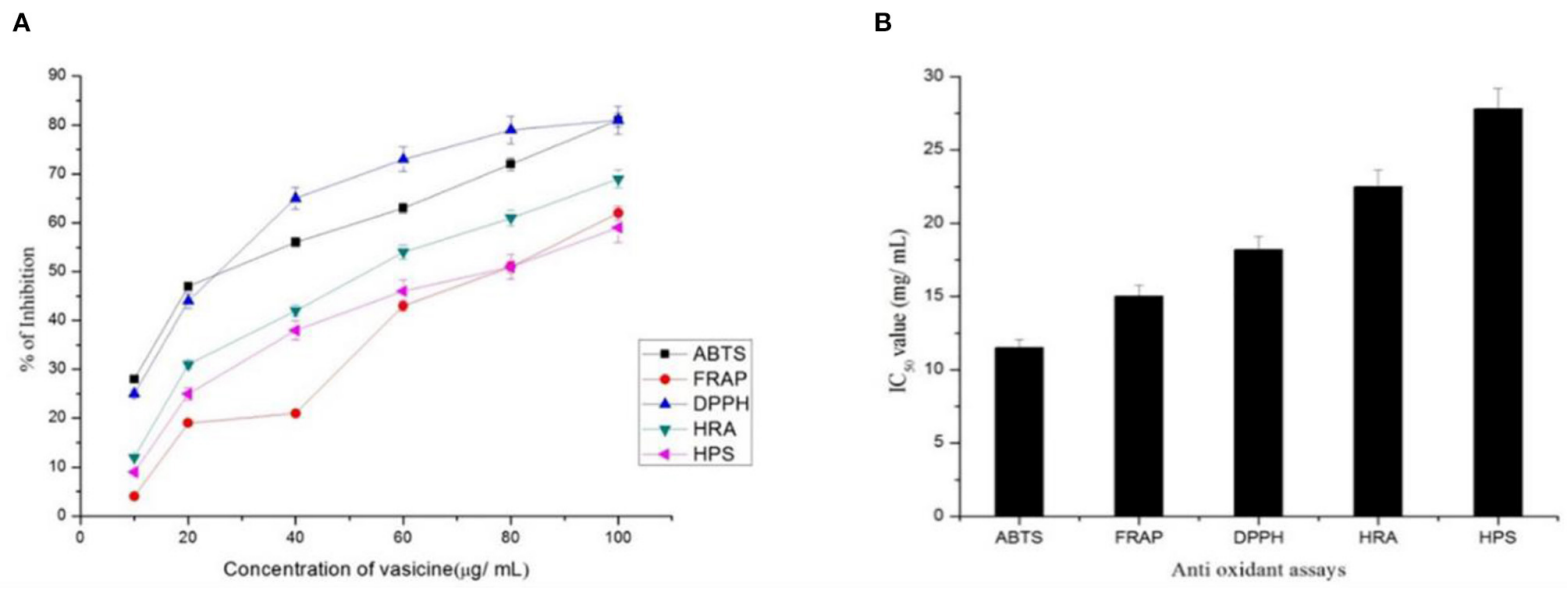
**(A)** Percentage inhibition and **(B)** IC_50_ value of vasicine in the antioxidant assay. ABTS, 2, 2'-azino-bis (3-ethylbenzthiazoline-6-sulphonic acid) scavenging Activity; FRAP, Ferric Reducing Antioxidant Power Assay; DPPH, 1,1 Diphenyl-2-picrylhydrazyl Radical Scavenging Activity; HRA, Hydroxyl Radical Scavenging Assay; HPA, Hydrogen Peroxide Assay.

Ascorbic acid was used in various concentrations as the standard drug to assess the scavenging property of vasicine. ABTS scavenging activity measures the relative capacity of antioxidant to scavenge the ABTS+ radicals of vasicine ranged from 28 to 75% (Figure 4) while that of the ascorbic acid ranged between 12 and 81% at a concentration of 100 mg/ml. The IC_50_ value of vasicine was calculated to be 11.5 μg/ml.

##### 3.2.2.2. Ferric reducing antioxidant power assay

Free radicals are generated due to the biochemical redox reactions occurring in human body as a part of normal cell metabolism. The oxidative stress is produced due to production and scavenging of free radicals, can cause many diseases such as cancer, arthritis, antheroclerosis, etc. In this study, vasicine was expressed in terms of FeSO_4_.7H_2_O equivalent. A correlation between different concentrations and the ferric reducing ability of vasicine was determined between the range of 10–100 μg/ml (Figure 4). The standard showed 82% inhibition at 100 μg/ml, while vasicine showed 62% of inhibition at 100 μg/ml. A similar study by Srinivasarao et al. (60) found increase in serum alkaline phosphatase in Swiss albino mice treated with vasicine, shows it a potential antioxidant. The IC_50_ value of vasicine was calculated to be 15 μg/ml.

##### 3.2.2.3. DPPH radical scavenging activity

At a concentration of 100 μg/ml, vasicine and the standard (ascorbic acid) showed 81 and 96% inhibition, respectively (Figure 4). At 100 μg/ml. The IC_50_ value of vasicine was calculated to be 18.2 μg/ml.

##### 3.2.2.4. Hydroxyl radical scavenging assay

Vasicine showed scavenging activity of about 12–69% inhibition while ascorbic acid showed 15–86% inhibition at 100 μg/ml (Figure 4). The IC_50_ value of vasicine was reported to be 22 μg/ml.

##### 3.2.2.5. Hydrogen peroxide assay

10–100 μg of vasicine exhibited 9–59% inhibitory activity against hydrogen peroxide. The scavenging action against hydrogen peroxide was induced by the same concentration of ascorbic acid (Figure 4). The scavenging activity on hydrogen peroxide at 100 μg of vasicine was lesser than ascorbic acid. The IC_50_ value of vasicine was calculated to be 27.8 μg/ml.

#### 3.2.3. Anti-inflammatory assay

##### 3.2.3.1. Proteinase inhibitory activity

**Figure 5:**
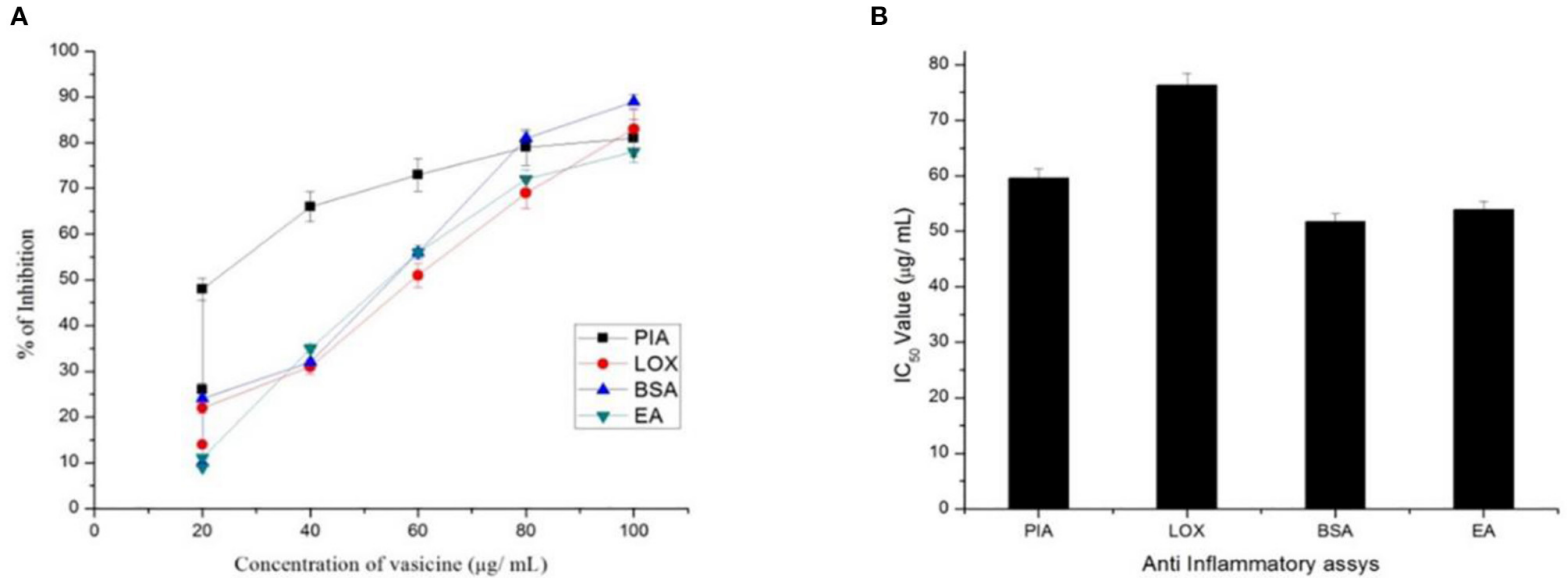
**(A)** Percentage inhibition and **(B)** IC_50_ value of vasicine in proteinase inhibitory activity (PIA), lipooxygenase inhibition assay (LOX), BSA using method (BSA), egg albumin method (EA).

The standard aspirin showed 79% inhibition (Figure 5). The activity was compared with diclofenac sodium (standard drug). The IC_50_ value of vasicine was 76 μg/ml.

##### 3.2.3.2. BSA method

A maximum percentage of inhibition of 82% was observed from the extracted vasicine (Figure 5). At 100 μg/ml, diclofenac sodium showed 42% inhibition. The effect of diclofenac sodium was found to be lesser when compared with that of the extracted vasicine. The IC_50_ value of vasicine was 51.7 μg/ml in comparison to the standard.

##### 3.2.3.3. Egg albumin method

This method was used to assess the anti-inflammatory effect of vasicine under *in vitro* conditions. Throughout the concentration range from 10 to 100 μg/ ml, the test extract exhibited inhibition of albumin denaturation (Figure 5). Vasicine and the standard drug (diclofenac sodium) showed inhibition at 81 and 89%, respectively. The IC_50_ value of vasicine was 53.2 μg/ml.

##### 3.2.3.4. Lipooxygenase inhibition assay

The IC_50_ value of vasicine against lipooxygenase was reported to be 76 μg/ml. The maximum percentage inhibition of 83% was observed with the extracted vasicine (Figure 5). Diclofenac sodium (standard) showed the inhibition of 82% at a concentration of 100 μg/ml.

#### 3.2.4. Antidiabetic activity

##### 3.2.4.1. α-amylase inhibition assay

**Figure 6:**
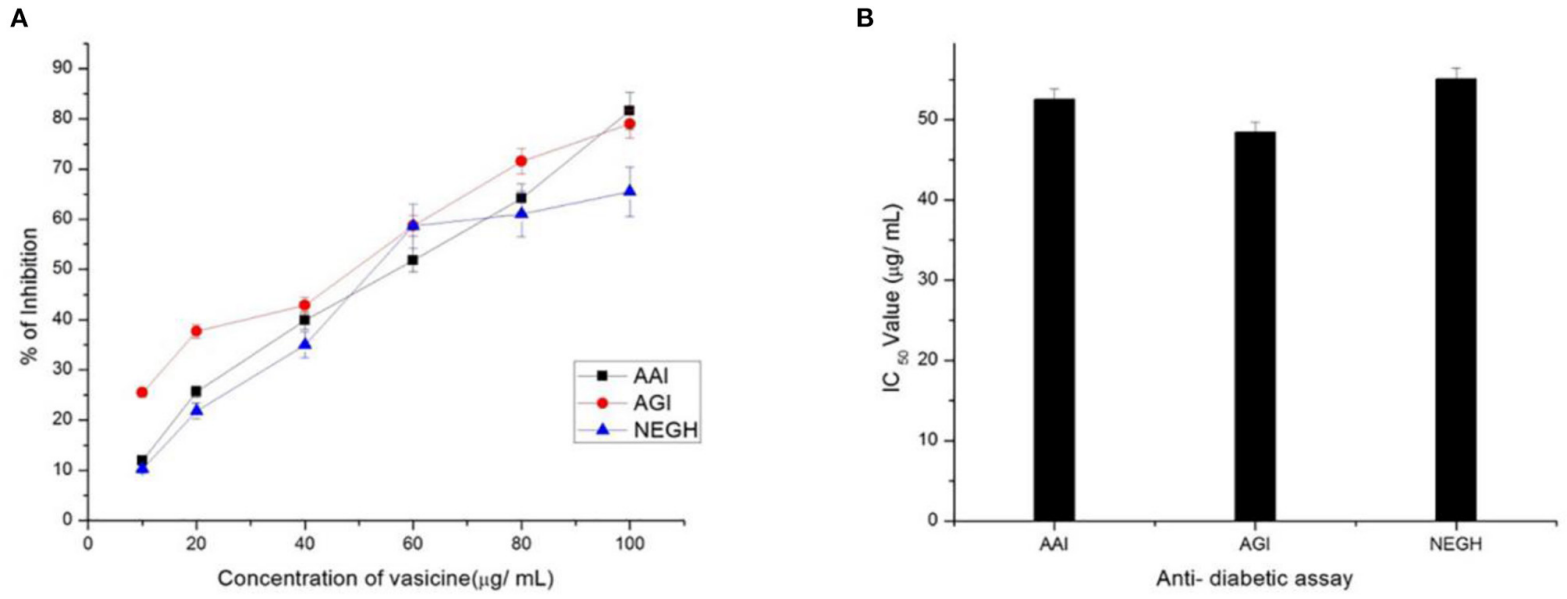
**(A)** Percentage inhibition and **(B)** IC_50_ Value of vasicine. AAI, α-amylase inhibition assay; AGI, α-glucosidase enzyme; NEGH, non-enzymatic glycosylation of hemoglobin assay.

α-amylase inhibitory assay revealed the potential of vasicine for the treatment of diabetes (Figure 6). The IC_50_ value of vasicine was calculated to be 47.6 μg/ml.

##### 3.2.4.2. α-glucosidase inhibition assay

In the present study, acarbose (positive control) inhibited α-glucosidase activity with an IC_50_ value of 49.68 μg/ml (Figure 6).

##### 3.2.4.3. Non-enzymatic glycosylation of hemoglobin assay

Our study showed an increase in glycosylation upon incubation of hemoglobin with glucose for 72 h (Figure 6).

#### 3.2.5. HIV-protease inhibition

**Figure 7:**
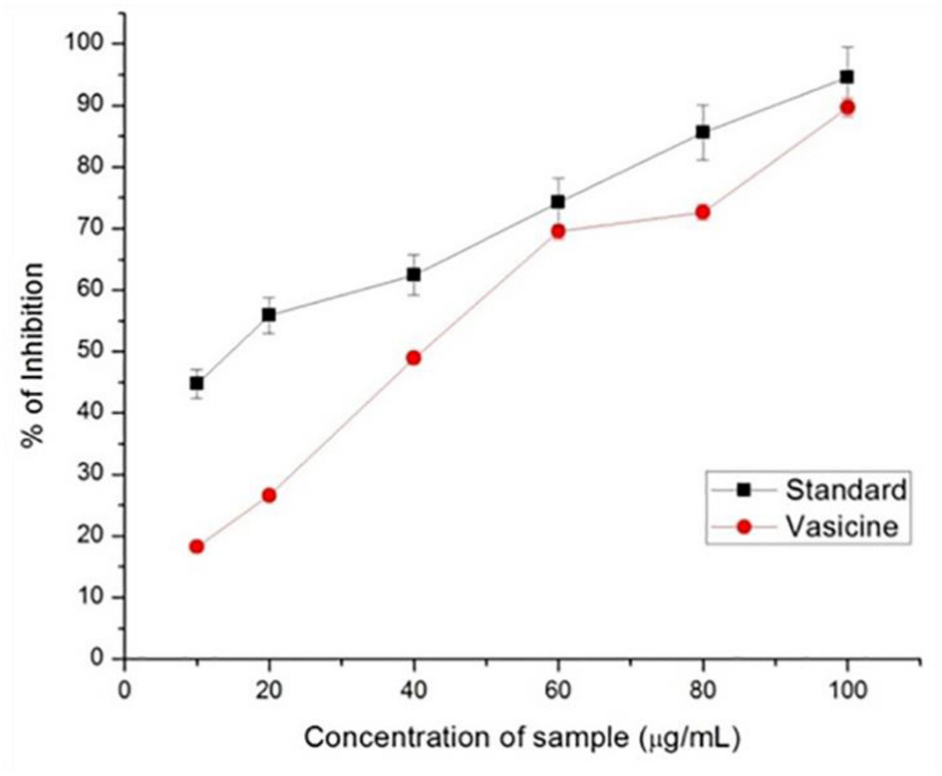
HIV-protease inhibitory activity of vasicine and the standard drug.

*A. vasica*aqueous extract showed 99% inhibition of pepsin. In this study, 89% inhibition of HIV-protease enzyme by vasicine was observed (Figure 7). The IC_50_ value of the vasicine was 38.5 μg /ml for HIV-protease.

#### 3.2.6. Anti-cancer activity

##### 3.2.6.1. Cytotoxicity assay by direct microscopic observationand MTT method

**Figure 8:**
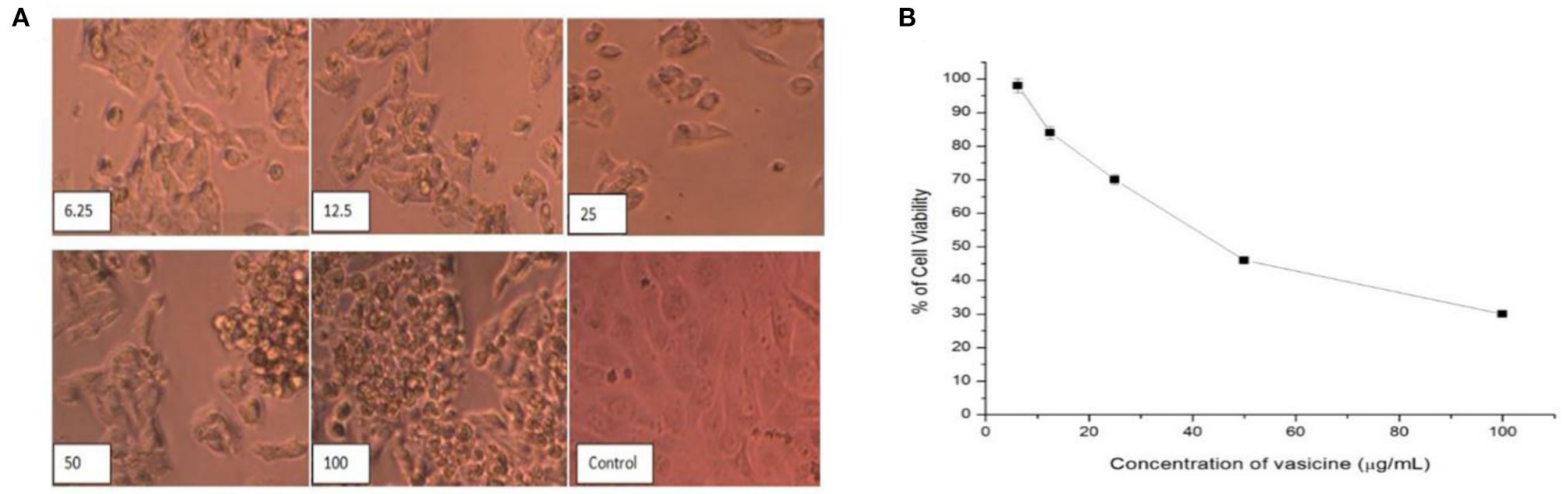
**(A)** Direct microscopic view on the anticancer activity of vasicine on lung cancer cell lines at various concentrations (6.25, 12.5, 25, 50, 100 μg/ml) in comparison with the control value. **(B)** Cell viability (%) in MTT assay (IC_50_ = 46.5 μg/ml).

Vasicine showed good anticancer activity against the lung cancer cell line (Figure 8A). As the concentration increases, there is an increase in cell growth inhibition. However, only 30.12% growth inhibition was observed at 100 μg/ml. The IC_50_ value of vasicine was < 100 μg/ml (Figure 8B). The results showed that vasicine had a very moderate anticancer activity.

##### 3.2.6.2. Cytotoxicity evaluation

**Figure 9:**
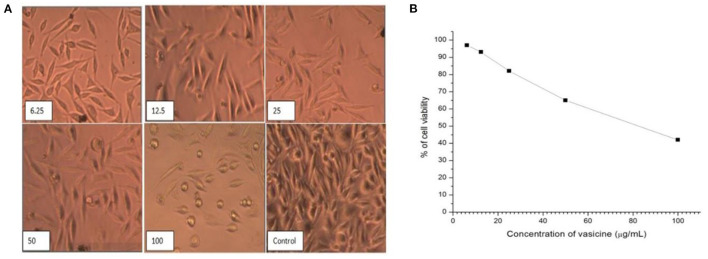
**(A)** Direct microscopic view of cytotoxic activity of vasicine at various concentrations (6.25, 12.5, 25, 50, 100 μg/ml) in comparison with the control value. **(B)** Cell viability (%) in MTT assay on Human Fibroblast Cell line (IC_50_ Value = 82.5 μg/ml).

Vasicine has a cytotoxic effect against fibroblast cell lines (Figure 9A). The IC_50_ of vasicine against fibroblast cell line was also higher than the IC_50_ value on lung cancer cells (Figure 9B).

#### 3.2.7. *In silico* studies

The binding affinity of vasicine toward the active site of each protein is given in Table 2. For comparative analysis, the binding affinity of the native ligand of each target protein is also provided in Table 2. Vasicine (-6.7 kcal/mol) showed a slightly lower binding affinity toward α-amylase than the native ligand (-8.7 kcal/mol). In case of α-glucosidase, vasicine (-7.6 kcal/mol) showed superior binding affinity than the native ligand (-6.1 kcal/mol). Vasicine (-7.4 kcal/mol) also exhibiteda better binding affinity for cyclooxygenase than the native ligand (-6.0 kcal/mol). Vasicine (-6.4 kcal/mol) showed a slightly better binding affinity for the epidermal growth factor and receptor than the native ligand (-6.3 kcal/mol). At a binding energy value of−6.4 kcal/mol, vasicine and the native ligand showed the same binding affinity toward HIV protease. Vasicine (-6.9 kcal/mol) showed a slightly lower binding affinity toward lipooxygenase than the native ligand (-7.4 kcal/mol).

**Table 2:** Binding affinity of vasicine in comparison to the native ligand of different proteins.

**Protein**	**Ligand**	**Binding energy (kcal/mol)**
α-amylase	Vasicine	−6.7
	Native ligand (3L9)	−8.7
α-glucosidase	Vasicine	−7.6
	Native ligand (GLC)	−6.1
Cyclooxygenase	Vasicine	−7.4
	Native ligand (SAL)	−6.0
Epidermal growth factor and receptor	Vasicine	−6.4
	Native ligand (NAG)	−6.3
HIV protease	Vasicine	−6.4
	Native ligand (478)	−6.4
Lipooxygenase	Vasicine	−6.9
	Native ligand (30Z)	−7.4

**Figure 10:**
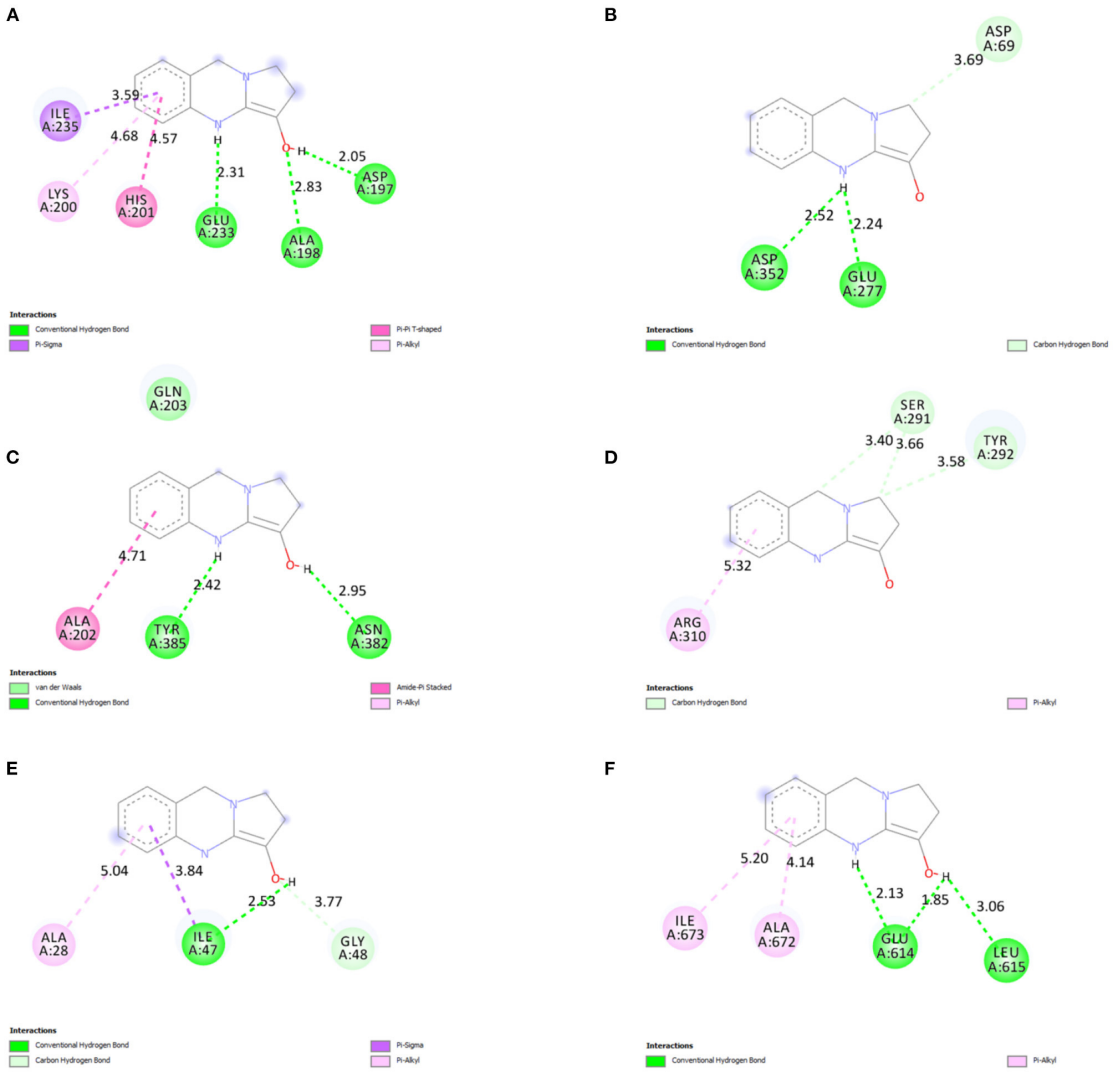
2D ligand interactions of vasicine with the different amino acid residues at the active binding sites of **(A)** α-amylase, **(B)** α-glucosidase, **(C)** cyclooxygenase, **(D)** epidermal growth factor and receptor, **(E)** HIV protease, and **(F)** lipooxygenase.

The 2D ligand interactions of vasicine with the target proteins can be visualized in Figure 10. Vasicine formed conventional hydrogen bonds [ASP197 (bond length = 2.05Å); ALA198 (bond length = 2.83Å); GLU233 (bond length = 2.31Å)] and hydrophobic interactions [LYS200 (bond length = 4.68Å); HIS201 (bond length = 4.57Å); ILE235 (bond length = 3.59Å)] with various amino acids at the active site of α-amylase (Figure 10A). Vasicine formed conventional hydrogen bonds [GLU277 (bond length = 2.24Å); ASP352 (bond length = 2.52Å)] and carbon-hydrogen bond [ASP69 (bond length = 3.69Å)] with different residues at the active site of α-glucosidase (Figure 10B). Vasicine formed conventional hydrogen bonds [ASN382 (bond length = 2.95Å); TYR385 (bond length = 2.42Å)] and hydrophobic interaction [ALA202 (bond length = 4.71Å)] with different amino acids at the active site of cyclooxygenase (Figure 10C). Vasicine formed carbon-hydrogen bonds [SER291 (bond lengths = 3.40Å, 3.66Å); TYR292 (bond length = 3.58Å)] and hydrophobic interaction [ARG310 (bond length = 5.32Å)] with different residues at the active site of epidermal growth factor and receptor (Figure 10D). Vasicine formed conventional hydrogen bond [ILE47 (bond length = 2.53Å)], hydrophobic interactions [ILE47 (bond length = 3.84Å); ALA28 (bond length = 5.04Å)], and carbon-hydrogen bond [GLY48, (bond length = 3.77Å)] with different residues at the active site of HIV protease (Figure 10E). Vasicine formed conventional hydrogen bonds [GLU614 (bond lengths = 1.85Å, 2.13Å); LEU615 (bond length = 3.06Å)] and hydrophobic interactions [ALA672 (bond length = 4.14Å); ILE673 (bond length = 5.20Å)] with various amino acids at the active site of lipooxygenase (Figure 10F). Drug-likeness study was carried out with the SwissADME tool. Vasicine followed all the rules and filters of Lipinski's rule of five, Ghose filter, Veber filter, and Egan filter. It showed one violation against Muegge filter as the molecular weight of vasicine was lesser than 200.

The study against *E. coli* and *Bacillus badius* confirms the antibacterial activity of purified vasicine. A comparative antioxidant study of 30 different plants extracts using ABTS+ radical scavenging assay. Radical scavenging activities are very important due to the deleterious role of free radicals in food and in biological systems. The result of study showed significant reduction in concentration of ABTS+ due to scavenging property of vasicine, which supports our study results with vasicine. Antioxidant activity reflects that vasicine inhibited ABTS (61–64). The activity increases with the increase in the concentration/ dose of the compound. In FRAP assay, the percentage inhibition increased with the increasing concentration of vasicine. With an increase in the concentrations of vasicine, an increase in the DPPH free radical scavenging activity of vasicine was observed. Researchers Ali et al. (65) investigated investigation of methanolic extract of *A. vasica* L. leaves by GC-MS and identified many bioactive constituents. A significant reduction in free radicals against DPPH was reported, which revealed the antioxidant potential of A. vasica leaves. Further, the scavenging action of vasicine on hydroxyl radical and H_2_O_2_ was observed to an appreciable extent and the inhibitory activity was increased with an increase in the concentration of vasicine. Denaturation of proteins is well-documented in inflammation (66). Vasicine was found to be effective in inhibiting heat-induced albumin denaturation. Inhibition of proteinase activity, BSA denaturation, egg albumin denaturation and lipooxygenase activity proved the anti-inflammatory activity of vasicine. Different studies on various plant extracts highlighted the role of vasicine to control hyperglycemia (67). Because of the inhibition of α-amylase and α-glucosidase vasicine could be used as a backup treatment for type-2 diabetes (68). As indicated by an increasing hemoglobin concentration in non-enzymatic glycosylation of hemoglobin assay, it can be observed that vasicine (in comparison to the standard drug) substantially inhibits hemoglobin glycosylation. The antidiabetic activity of vasicine has been reported for first time with this approach. The purified form of vasicine showed higher inhibition of HIV-protease. The present study confirmed that vasicine is an efficient inhibitor of HIV-protease. *A. vasica* was traditionally used to treat lung cancers *via* oral treatment. In the present study, a microscopic view of the MTT assay inferred that the cells were detached from the substance and they form a group in the medium. Vasicine exhibited cytotoxic effect against the lung cancer cell line. More than 50% inhibition of cell growth was observed. The cytotoxic effect was also observed against fibroblast cell lines. From this microscopic view of the MTT assay, inferred that the cells were connected to the substance and they are cannot form a group in the medium at a lower concentration. The binding energy values obtained from the molecular docking studies revealed that vasicine has the affinity to bind to the active binding sites of all the target proteins. Drug-likeness was satisfactory for vasicine. Drug-likeness parameters such as Lipinski's rule of five, Ghose filter, Veber filter, and Egan filter were within the acceptable limit.

## 4. Conclusion

The study reports that vasicine isolated from *A. vasica* leaves is a potent bioactive compound with a potential for the treatment of microbial infection, oxidative stress, inflammation, diabetes, viral infections, and cancer investigated by various *in vitro* studies. *In silico* studies reveals that vasicine have the inhibitory properties against HIV-protease, α-amylase, α-glucosidase, cyclooxygenase, lipooxygenase and epidermal growth factor receptor. The present study finally confirms the potential of vasicine as a bioactive compound isolated from *A. vasica* having therapeutic usefulness in many human diseases. The study further validates the traditional importance of *A. vasica* leaves in the management of various human ailments through an array of *in vitro* and *in silico* studies.

## Data availability statement

The original contributions presented in the study are included in the article/supplementary material, further inquiries can be directed to the corresponding authors.

## Author contributions

MR and SV: conceptualization, methodology, software, investigation, writing—original draft, review and editing, resources, and supervision. SV, SA, JK, HA, AA-S, and MK: validation and formal analysis. SA, JK, HA, AA-S, and MK: funding acquisition. MR: critical analysis and final draft-review and editing. All authors contributed to the article and approved the submitted version.
